# Application of *Ligilactobacillus salivarius* SP36, a Strain Isolated from an Old Cheese Seal, as an Adjunct Culture in Cheesemaking

**DOI:** 10.3390/foods13142296

**Published:** 2024-07-21

**Authors:** Ramón Arias, Claudio Alba, Javier Calzada, Lorena Jiménez, Leónides Fernández, Marta Ávila, Mario Roman, Juan Roman, Juan M. Rodríguez, Sonia Garde

**Affiliations:** 1 Centro Regional de Selección y Reproducción Animal (CERSYRA), Instituto Regional de Investigación y Desarrollo Agroalimentario y Forestal de la Junta de Comunidades de Castilla-La Mancha (IRIAF), CERSYRA Valdepeñas, 13300 Ciudad Real, Spain; ljimenez@quesomanchego.es; 2 Department of Nutrition and Food Science, Complutense University of Madrid, 28040 Madrid, Spain; c.alba@ucm.es (C.A.); jmrodrig@ucm.es (J.M.R.); 3 Departamento de Tecnología de Alimentos, Instituto Nacional de Investigación y Tecnología Agraria y Alimentaria (INIA-CSIC), Carretera de La Coruña km 7, 28040 Madrid, Spain; jcalzada@inia.csic.es (J.C.); arribas@inia.csic.es (M.Á.); 4 Department of Galenic Pharmacy and Food Technology, Complutense University of Madrid, 28040 Madrid, Spain; leonides@ucm.es; 5 QUALIAM s.l., Velazquez 119, 28003 Madrid, Spain; mroman@qualiam.es (M.R.); juanromanacacio@gmail.com (J.R.)

**Keywords:** traditional cheese, *Ligilactobacillus salivarius*, adjunct culture, sensory properties

## Abstract

Adjunct cultures originating from artisanal cheese environments may play an important role in recreating and developing traditional cheese flavours, thanks to their enzymatic activities, involved in different metabolic pathways that occur during cheese ripening. In this work, *Ligilactobacillus salivarius* SP36, a strain isolated from an old cheese seal, was added as an adjunct culture to the cheese’s raw milk, and its effect on the microbiological, physical–chemical and sensory characteristics of the cheese was studied. The use of *L. salivarius* SP36 in cheese manufacturing had no significant (*p* > 0.05) effect on the cheese microbiota, gross composition (fat percentage, protein, total solids, moisture and NaCl concentration), colour or texture of the cheese. However, *L. salivarius* SP36 increased (*p* < 0.01) the formation of 25 volatile compounds, including 10 esters, 1 aldehyde, 8 alcohols and 6 carboxylic acids. In addition, cheeses made with *L. salivarius* SP36 received higher scores (*p* < 0.01) for aroma intensity and quality than control cheeses. *L. salivarius* SP36 proved to be a good candidate as an adjunct culture for cheesemaking, since it improved the cheese flavour by making it more intense and recovering typical sensorial notes of traditional cheeses.

## 1. Introduction

The control on how the microbial communities evolve during cheese manufacturing and ripening is essential for modern cheesemaking [1,2]. As a consequence, cheese companies relies in the inoculation of milk with cultures composed of well-selected microorganisms, including starter cultures, when they are required to catalyse the fast initial acidification, and adjunct cultures, when they exert a noticeable influence on maturing activities, including the development of the sensorial properties that may characterize a certain type of cheese [3,4]. Starter cultures usually contain strains of *Lactococcus lactis* and/or *Streptococcus thermophilus*, while adjunct cultures may include a wide variety of species belonging to some of the genera in which the genus *Lactobacillus* was recently reclassified [5] and other lactic acid bacteria (LAB).

The microbiota of traditional raw-milk cheeses has gained a renewed interest in the search for LAB strains with relevant genomic and phenotypic traits for food and biotechnological applications [6,7,8]. In the dairy context, such strains could complement current commercial strains in order to recover or preserve the typical sensorial properties of the traditional cheeses or to provide novel dairy products [9,10,11]. Improvement of the organoleptic features of a given cheese may increase consumer’s attraction and commercial value.

Many traditional cheeses have disappeared in recent decades, and many of those surviving at present are still manufactured by small familiar companies on a small scale for local or tourism-related sales. The number of those that do not employ starter cultures (and, therefore, which maturation relies on indigenous microorganisms, often applied using back-slopping approaches) is declining rapidly. In contrast, large-scale cheese companies employ pasteurized milk inoculated with starter cultures that repress the growth of the indigenous microorganisms that may arise from milk or from the dairy environment. Therefore, the biodiversity of the microbiota of industrial cheeses is reduced and often limited to or overwhelmingly dominated by the inoculated species and strains [12]. In turn, it leads to a loss of some of the organoleptic qualities linked to traditional cheeses. It has been repeatedly suggested that isolating and characterizing strains from naturally fermented traditional cheeses may help to provide new cultures that could be used by either small/traditional and large/industrial dairy companies to produce more safe or improved cheeses, respectively [13,14].

Recently, a *Ligilactobacillus salivarius* strain (*L. salivarius* SP36) was isolated from an old seal used to label the cheeses made by a producer living in a Pyrenean village [15]. In vitro assessment showed that the strain was safe and had some functional properties, including antimicrobial activity against cheese-related spoilage and foodborne microbes. The study of its genome revealed its potential for the production of organoleptic-related compounds. In this context, the objective of this work was to compare the performance of pressed cheese elaborated with a commercial starter culture and that of equivalent cheeses in which *L. salivarius* SP36 was added as an adjunct culture in addition to the starter culture.

## 2. Materials and Methods

### 2.1. Cheese Manufacture

Two batches of experimental cheeses were made in duplicate, using in the first type of preparation a commercial starter (CS) and in the second type the same commercial starter together with the strain *L. salivarius* SP36 as an adjunct culture (LS). The pressed cheeses were made with 80 litres of raw sheep bulk-tank milk, which was transported in less than 1 h and under refrigeration conditions (6 °C) from the farm to the CERSYRA cheese plant. In the case of the CS cheeses, the milk in agitation in the curd vat at 30 °C was inoculated with a commercial starter culture containing *Lactococcus lactis* subsp. *lactis*, *Lactococcus lactis* subsp. *cremoris*, *Lactococcus lactis* subsp. *lactis* biovar. *diacetylactis* and *Streptococcus thermophilus* (CHOOZIT MA4001 LYO 5 DCU, Danisco, France) at a dose of 4 DCU/80 L milk. In the case of the LS cheeses, the milk was inoculated with the same commercial starter culture at the same dose and at the same temperature (30 °C) but, also, with *Ligilactobacillus salivarius* SP36, a strain previously isolated from an old cheese seal [15] (50 g containing 9 log cfu/g in 80 L of milk). After 30 min, rennet was added (185 IMCU/mL, Laboratorios Arroyo, Santander, Spain), following the routine methodology of the cheese industry (30 °C, 40 min). Next, the curd was cut and mixed (30 °C, 10 min), and the temperature was gradually increased from 30 °C to 36 °C for 15 min. After placing the curd in the 3 kg cheese moulds, pressing was carried out by exerting a pressure of 2–3 kPa for 5–6 h at 15 °C and turning the cheeses every hour up to pH < 5.4. The cheeses were salted in a 20% NaCl brine pH = 5.2–5.4) for 15 h at 8 °C. The average cheese yield was 0.23 kg/L of milk. The cheeses were dried at 7 °C with a relative humidity of 90% for 10 d, and ripening was performed at 10 °C with a relative humidity of 80% for 240 d. In all the elaborations, there was a similar decrease in the weight (~750 g; ~20%) between 1 and 240 days after manufacture. Cheeses were analysed at 1, 60, 120, 180 and 240 d, classifying them as young (1 d), hard (60 and 120 d) and old cheeses (180 and 240 d) according to Spanish legislation.

### 2.2. Physico-Chemical and Microbiological Analyses of Milk

The physico-chemical characteristics (percentage of fat and protein) of the bulk-tank milk were analysed using MilkoScan equipment (Foss Electric, Hillerød, Denmark) and pH with pHmeter basic Crison (Barcelona, Spain). The microbiological composition of milk was assessed using PCA plates (Oxoid, Basingstoke, UK) for mesophilic bacteria counts and MRS plates (Oxoid) for lactic acid bacteria. Plates were incubated at 32 °C for 48 h.

### 2.3. Physico-Chemical and Microbiological Analyses of Cheeses

Cheeses were sampled at days 1, 60, 120, 180 and 240. The physico-chemical parameters (fat, protein, total solids and moisture, as well as NaCl concentration) were measured in duplicate with the near-infrared spectroscopy FOOD SCAN LAB (Foss Electric, Hillerød, Denmark), provided with ISISCAN 4.6 software. Each sample consisted of 50 g of crushed cheese collected from the central area of the cheese. At each sampling time, cheese aliquots were added to a sterile peptone solution and homogenized in a stomacher (IUL Instrument, Barcelona, Spain). Proper decimal dilutions of the homogenized samples were seeded in duplicate in MRS plates supplemented with L-cysteine (0.05%, *w*/*v*). MRS plates were incubated aerobically at 30 °C for 48 h for the selective enumeration of *L. lactis* and anaerobically at 37 °C for 48 h for the selective enumeration of *Lactobacillus* (*sensu lato* [5]). PCA plates were incubated at 42 °C for 48 h for the selective enumeration of *S. thermophilus*. Microbial counts were expressed in log_10_ cfu/g. Cheese pH was also measured at each sampling point.

Subsequently, a metataxonomic analysis of cheeses samples collected at days 1, 120 and 240 was performed. DNA was extracted as described previously [16]. A dual-barcoded 2-step PCR reaction was conducted to amplify a fragment of the V3–V4 hypervariable region of the bacterial 16S ribosomal RNA (rRNA) gene. Equimolar concentrations of the universal primers S-D-Bact-0341-b-S-17 (ACACTGACGACATGGTTCTACACCTACGGGNGGCWGCAG) and S-D-Bact-0785-a-A-21 (TACGGTAGCAGAGACTTGGTCTGACTACHVGGGTATCTAATCC) were used. Barcodes used for Illumina sequencing were appended to the 30 and 50 terminal ends of the PCR amplicons to allow for the separation of forward and reverse sequences. A bioanalyser (2100 Bioanalyzer, Agilent, Santa Clara, CA, USA) was used to determine the concentration of each sample. Barcoded PCR products from all samples were pooled at approximately equimolar DNA concentrations and run on a preparative agarose gel. The correctly sized band was excised and purified using a QIAEX II Gel Extraction Kit (Qiagen, Hilden, Germany) and then quantified with PicoGreen (BMG Labtech, Jena, Germany). Finally, 1 aliquot of pooled, purified and barcoded DNA amplicons was sequenced using the Illumina MiSeq pair-end protocol (Illumina Inc., San Diego, CA, USA) at the facilities of the Scientific Park of Madrid (Spain). The sequences analysed in this study are available in the BioSample database of the National Center for Biotechnology Information under accession number PRJNA1081834. The amplified fragments and results were taxonomically analysed using the Illumina™ software according to the manufacturer’s guidelines and pipelines (version 2.6.2.3).

Taxonomic affiliation for individual amplicon sequence variants (ASVs) was ascertained through the q2 feature classifier, utilizing a naïve Bayes taxonomic classification schema against the SILVA 138.1 reference database. A matrix detailing ASVs specific to each respective sample was systematically generated. The subsequent normalization of bacterial taxa abundances was performed utilizing the total sum scaling (TSS) method, wherein each discrete ASV count was divided by the cumulative library size, thereby rendering the relative proportion of individual ASV counts per sample. For the examination of alpha diversity, both the Shannon and Richness diversity indices were employed, facilitated through the vegan package within R Statistical Computing Environment (Version 2.5.6).

### 2.4. Colour and Texture Analyses of Cheeses

The colour values of the cheeses were measured using the CIELAB colour space. Variables included lightness (L*), red/green value (a*) and blue/yellow value (b*), which were measured using a spectrophotometer CM-2300d (Konika Minolta Sensing, Inc., Tokyo, Japan). Texture profile analyses (TPAs) were performed to determine the values of hardness, adhesiveness, cohesiveness, gumminess, chewiness, springiness and resilience, following the method proposed by Romeith et al. [17]. A food texture analyser equipped with a 30 cm diameter cylindrical probe was used (Shimadzu, Kyoto, Japan). These internal cheese samples had a cylindrical shape with 11 mm of diameter and 15 mm of height and, immediately after their collection, were covered with plastic film to prevent desiccation and allowed to warm at room temperature (20 ± 2 °C) for 30 min before analysis. These analyses were carried out in quadruplicate.

### 2.5. Analysis of Volatile Compounds

Cheese pieces wrapped in aluminium foil were vacuum packed and frozen at −40 °C at 60, 120, 180 and 240 d of ripening, until analysis. Volatile compounds of cheeses were extracted by automated solid-phase microextraction (SPME) and analysed by gas chromatography–mass spectrometry (GC-MS) as described previously [18]. All determinations were carried out in duplicate. The detection, identification and semi-quantification of volatile compounds were carried out as described by Garde et al. [19]. Mass detection was performed in the scan mode, from 33 to 300 amu at 5.16 scan/s, and ionization by EI at 70 eV. Data were collected with the HP ChemStation program (G2070-90107), and volatile compounds were identified by comparison of spectra with the Wiley 275 library and by comparison of their retention times with authentic standards (Sigma Chemical Co., St. Louis, MO, USA). Relative abundances of compounds were expressed as percentages of their peak areas on the peak area of the internal standard cyclohexanone.

### 2.6. Sensory Analysis of the Cheeses

Two different sensorial analyses were performed during this work. The first one was carried on cheeses from 60 days of ripening, following the methodology of the ISO Standard Nº 13299:2016 [20] and PDO Manchego Cheese Sensory Guide (Procedure PAS-02). On the other hand, a descriptive test was developed for cheeses based on the guidelines of hard and semi-hard cheeses given by Bérodier et al. [21]. Eight trained panelists evaluated the cheeses at 60, 120, 180 and 240 days of ripening for quality (overall acceptance) and intensity (overall intensity) of odour, aroma and taste, and for texture and colour quality on a 0–10-point scale. Panelists were also asked to assign a score on a 1- (very slight perception) to 7-point (very intense) scale to the taste attributes “sour”, “bitter”, “sweet”, “salty” and “umami”, and separately for odour and aroma attributes belonging to six families, namely, “lactic” (including the descriptors “milky”, “buttery”, “yogurt-like” and “cheesy”); “vegetal”; “fruity-flowery” (including the descriptors “fruity” and “nutty”); “toasted” (including the descriptors “caramel” and “toasted”); “animal” (including the descriptor “sheepy”); and “others” (including the descriptor “pungent”). Informed consent was obtained from the sensory participants, which were apprised about the samples, protocols and their rights as participants.

Prior to sensory evaluation, cheeses were held for 3 h at 20 to 22 °C. Then, they were cut in representative triangular slices (15–20 g) after removing the rind. Slices of the two cheeses per session from each of the two vats manufactured on the same day, coded with random three-digit numbers, were presented to panelists in random order. Bread and water were provided to cleanse the mouth between cheeses.

### 2.7. Statistical Analysis

Cheeses were manufactured in duplicate experiments on different days, and all determinations were performed in duplicate. Statistical treatment of data was performed by SPSS software (IBM SPSS Statistics version 29, IBM Corp., Armonk, NY, USA). Data were analysed by ANOVA using a general linear model, and comparison of means was carried out with a least significant difference (LSD) test.

## 3. Results and Discussion

### 3.1. Compositional and Microbiological Characteristics of Milk

The bulk-tank milk used had the following compositional characteristics: pH = 6.51, fat = 8.57% and protein = 5.85%. Counts on PCA and MRS agar plates were <5 log cfu/mL.

### 3.2. Microbiological Characteristics of the Cheeses

The populations of *L. lactis* and *S. thermophilus* decreased gradually over the ripening time, but no differences (*p* > 0.05) were found between the CS and the LS cheeses in relation to their counts throughout the ripening period (Table 1). The concentration of lactobacilli decreased significantly between day 1 and day 60 of ripening, from 8.72 to 6.54 log cfu/g while it remained stable from day 60 to the end of ripening, and no significant differences were found throughout the rest of ripening. 

In relation to the metataxonomic analysis of three CS cheeses at days 1, 120 and 240 and three LS cheeses at the same sampling times, the sequencing of the six cheese samples yielded 1,502,505 high-quality reads (median = 237,910.5 reads/sample, ranging from 200,787.0 to 284,112.8 reads/sample). The two diversity indices evaluated in this work (Shannon and Simpson indices) increased over time in both types of cheeses. For CS cheeses, the Shannon indices were 0.53, 0.66 and 0.99 at days 1, 120 and 240, respectively, while the values at the same sampling times where 0.60, 0.77 and 0.97 for LS cheeses. In relation to the Simpson index, the values were 0.33, 0.32 and 0.54 at days 1, 120 and 240, respectively, among the CS samples and 0.35, 0.36 and 0.49, respectively, among the LS ones. No differences were found when CS and LS samples were compared within each sampling time, but there was a significant increase for both diversity indices and both types of cheeses when samples of days 1 and 240 were compared (*p* < 0.01). It has been previously found that diversity in the microbiota of cheeses is a major driver in the organoleptic characteristics found in raw-milk cheeses [22].

The genera detected in the metataxonomic analysis of the samples are shown in Table 2. Among them, the genus Lactococcus was the most abundant in both cheese types although its concentration decreased from day 1 to day 240. The genus Streptococcus was the second genus in order of abundance in both cheese types at days 1 and 120, and its percentage also decreased throughout the ripening period. The genus *Ligilactobacillus* was present from the beginning in the LS cheeses and its relative abundance increased during ripening, probably because of the decrease in the relative abundances of genera *Lactococcus* and *Streptococcus*. In contrast, *Ligilactobacillus* sequences were sparsely detected in CS cheeses. Interistingly, *Lactiplantibacillus* sequences, probably with an environmental origin, were not detected on day 1 in any of the cheese types but its abundance increased over time, and in fact, it was the second most abundant genus in both cheese types at the end of the ripening period. *Lactiplantibacillus plantarum* and *Lactiplantibacillus pentosus* are common and often dominant members of the non-starter LAB communities in cheeses [12,23,24]. These microbes are present at low or even not detectable concentrations in the curd, but their levels increase notably during ripening [24], which is in agreement with the results of this study. Despite the great abundance of *Lactiplantibacillus* sequences in both cheeses, these microorganisms were not detected in the microbiological analyses, probably because they do not grow in the medium used and/or the conditions tested.

### 3.3. Compositional Characteristics of Cheeses

The physical–chemical characteristics of both cheese types were within the ranges established in the regulation of the differentiated quality figure for Manchego cheese [25]. No differences in the percentage of fat, protein, moisture and amount of NaCl were observed when both groups (LS and CS) were compared (Table S1). With respect to pH, the values were significantly higher in LS cheeses on 1 day (5.38 vs. 5.09) and on day 60 (5.23 vs. 5.16), but no differences were detected in the remaining sampling times (120, 180 and 240 days) (*p* > 0.05). There was no significant difference in colour parameters between both types of cheeses although the values of red index (a*) and yellow index (b*) were higher in the LS cheeses.

### 3.4. Textural Properties of Cheeses

The texture profiles of the cheeses at different stages of ripening are presented in Table S2. No significant differences were observed for these parameters between the CS and LS cheeses, although the evolution of descriptors was slightly less marked in the LS ones. The texture of the cheeses changed as the ripening process progressed, due to several biochemical events, as indicated by Lucey et al. [26]. Hardness of the cheeses was lower on day 1 than on day 60 of ripening for the two types of cheeses, with significant differences regarding LS cheeses, although the values tended to stabilize from then to day 240 of ripening. Adhesiveness also increased as cheese ripening progressed. In contrast, cohesiveness decreased with time. Gumminess of the cheeses decreased significantly as ripening progressed. This behaviour is similar to that described by Salvador et al. [27] in pressed cheeses. Chewiness showed significant differences among CS cheeses throughout ripening, with a maximum level at 60 days, then decreasing and increasing again at the end of ripening. On the contrary, the chewiness in LS cheeses evolved with a gradual decrease throughout the ripening process. Finally, springiness and resilience of cheeses shared similar values in the samples obtained up to 120 days of ripening in both cheese types but then they increased slightly until the end of ripening, with significant differences when CS cheeses at day 240 were compared with the same CS cheeses in the previous sampling times.

### 3.5. Analysis of Volatile Compounds

A total of 82 volatile compounds were identified by SPME/GC-MS in the cheeses, including 5 hydrocarbons, 5 sulphur compounds, 4 aldehydes, 9 ketones, 11 esters, 20 alcohols, 6 benzene compounds and 12 carboxylic acids. The use of *L. salivarius* SP36 as an adjunct culture significantly increased (*p* < 0.01) the formation of 25 of these volatile compounds (Table 3, Table 4 and Table 5).

*L. salivarius* favoured the formation of 6 ethyl-, 2 propyl-, 1 butyl- and 1 branched esters and their relative abundances increased significantly (*p* < 0.001) during LS cheese ripening (Table 3). The biosynthesis of aroma-active esters in dairy systems proceeds through two enzymatic mechanisms: esterification, a reaction in which esters are formed from alcohols and carboxylic acids, and alcoholysis, a transferase reaction in which fatty acyl groups from acylglycerols and acyl-CoA derivatives are directly transferred to alcohols. Evidence has been provided that esterases of lactic acid bacteria catalyse not only the hydrolysis of milk fat glycerides to release free fatty acids but also the synthesis of esters from glycerides and alcohols via alcoholysis in cheese [28]. The observed differences in ester concentration among cheeses could be related to the different lactic acid bacteria compositions; it has been described that *L. salivarius* strains isolated from human milk displayed esterase activities on most assayed substrates when tested for dairy technological properties [29]. Esters, which usually increase during cheese ripening, have a very low perception threshold, playing an important role in the aroma profiles of different cheese varieties [30,31,32,33,34]. Most esters encountered in cheese are described as having sweet, fruity and floral notes [30,34].

Cheeses made with the *L. salivarius* strain showed higher concentrations of 3-methyl butanal than control (CS) cheeses (Table 4). This branched-chain aldehyde originates from leucine by transamination, leading to an intermediate imide that can be decarboxylated, or by Strecker degradation, and has been identified as a potent odourant in several cheese varieties [30]. A high aminopeptidase activity has been described in *L. salivarius* strains, including Leu-pNA as a substrate [29]. 3-Methyl butanal presents a green malty odour, but at low concentration, the odour becomes fruity and rather pleasant.

The levels of 1-propanol, 1-hexanol, 1-octanol, 1,2-propanediol and 2-furanmethanol were significantly (*p* < 0.01) higher in cheeses made with *L. salivarius* SP36 throughout ripening (Table 4). However, the levels of ethanol and 1-butanol were only higher (*p* < 0.01) at the end of ripening, while the levels of 2-pentanol were higher (*p* < 0.01) at 60 and 120 d in cheeses made with *L. salivarius*. The relative abundances of all these alcohols increased (*p* < 0.01) with cheese age. Many metabolic pathways are involved in the biosynthesis of the alcohols commonly found in cheese, such as lactose metabolism, methyl ketone reduction, amino acid metabolism, as well as degradation of linoleic and linolenic acids [30]. In general, primary alcohols have a high perception threshold, so scarcely contribute to the aroma of cheese. However, they are the limiting factor in ester formation in hard cheeses [34]. With respect to 1,2-propanediol, Fröhlich-Wyder et al. [35] described the production of this compound when *Lactobacillus buchneri* and *Lactobacillus parabuchneri* were used as adjunct cultures in the manufacture of model cheese. On the other hand, 2-furanmethanol has been found to contribute to the nutty and roasted aroma of Parmigiano-Reggiano cheese [36].

The use of *L. salivarius* SP36 as an adjunct culture in cheese manufacturing also promoted the formation of six carboxylic acids at the end of ripening (*p* < 0.01) (Table 5). As with the rest of volatile compounds mentioned above, the concentrations of these acids increased (*p* < 0.001) with cheese age. During cheese ripening, most of the acids having between two and six carbon atoms originate from the degradation of lactose and amino acids, but they can also be derived from ketones, esters and aldehydes by oxidation [30]. Short-chain carboxylic acids have low perception thresholds and are major odourants of different cheese varieties. Propanoic acid has a typical vinegar odour, and pentanoic and hexanoic acids contribute significantly to the aroma of aged cheeses [31,32,33]. On the other hand, branched-chain carboxylic acids such as 2-methyl propanoic acid and 2- and 3-methyl butanoic acids are characteristic odour impact compounds of goat and sheep cheeses. They derived from valine, isoleucine and leucine, respectively [30].

To our knowledge, this is the first time that a *L. salivarius* strain has been used as an adjunct culture in the manufacture of sheep cheeses. In a previous work, the potential of two human milk *L. salivarius* strains in fresh model cheeses to develop a probiotic cheese was evaluated [37]. However, the effect of these strains on the volatile profile of cheeses was limited. More recently, the use of *L. salivarius* AR809 as an adjunct in the manufacture of Monascus-ripened cheese (a mould-ripened cheese) significantly promoted the formation of alcohols, acids and ketones but reduced the formation of certain ethyl esters [38].

### 3.6. Sensory Evaluation

The evaluation of the sensorial properties of the cheeses from 60 days of ripening was initially performed following the methodology of the ISO Standard Nº 13299:2016 and the PDO Manchego Cheese Sensory Guide (Procedure PAS-02). This evaluation concluded that, in both types of cheeses, the external descriptors (shape, physical integrity, colour of the rind, uniformity of the engraving of the pleita, uniformity of the engraving of the flower, hardness of the rind, degree of rind distinction, uniformity of the colour of the cheese paste, colour of the cheese paste, distribution and size of the eyes, and integrity of the paste) as well as the internal descriptors (odour, texture and flavour) were within the ranges typical of Manchego cheese. To the appreciation of the trained panelists, both cheeses had similar sensorial properties overall (Figure S1), but LS cheeses had more complex flavours than CS ones. This is a logical finding since CS cheeses were made with a starter culture that is well tested by dairy companies, including those producing Manchego cheeses, and selected to provide cheese uniformity, which is an essential issue for consumers’ reliability. Therefore, it is not strange that the addition of a *L. salivarius* strain with the ability to produce sensorial-related compounds led to the appreciation of “wilder” flavours; spicier touches; and greater firmness, microstructure and friability (Figure S1).

Subsequently, the cheeses were also evaluated and compared using a descriptive test based on the guidelines of Bérodier et al. [21]. The cheeses were evaluated for taste, odour and aroma quality and intensity, and for texture and colour quality, throughout ripening (Table 6). The taste intensity and aroma intensity, and texture quality (*p* < 0.01) of the cheeses increased significantly with cheese age (*p* < 0.001), whereas taste quality, odour quality, aroma quality and colour quality, and odour intensity remained stable. Odour intensity and quality scores were higher in cheeses made with the *L. salivarius* strain, but significant differences were not detected (*p* > 0.01). However, the panelists gave significantly higher aroma intensity scores to cheeses made with *L. salivarius* throughout ripening (*p* < 0.01) (Table 6). Aroma quality scores were also higher for cheeses made with *L. salivarius* and were significantly higher after 120 and 240 d of ripening (*p* < 0.01). The higher aroma intensity and aroma quality scores obtained for cheeses made with the *L. salivarius* strain are in accordance with the higher levels of volatile compounds detected in these cheeses (Table 3, Table 4 and Table 5).

In addition, cheeses made with *L. salivarius* SP36 obtained significantly higher “sheepy”, “pungent”, “fruity” and “nutty” odour and aroma scores than control cheeses (*p* < 0.001). The higher “pungent” odour and aroma scores obtained by cheeses made with *L. salivarius* SP36 could be related to the higher levels of carboxylic acids found in these cheeses since many of them have been associated with “pungent” odours notes [30]. The increase in “fruity” and “nutty” aroma scores in cheeses including the *L. salivarius* strain could be associated with the higher levels of esters and furanmethanol found in these cheeses, respectively. Fruity and nutty flavour notes have been described for these compounds, respectively [30,36]. In a previous study, the use of two human milk *L. salivarius* strains in fresh cheeses did not have a relevant impact in the sensory quality and acceptance of the cheese, which was in agreement with their limited effect on their volatile profiles [37]. In contrast, the use of *L. salivarius* AR809 as an adjunct in the manufacture of Monascus-ripened cheese had a positive effect on sensory properties [38]. This strain significantly promoted the formation of several volatile compounds in cheese.

Taste intensity and quality scores were also higher for cheeses made with *L. salivarius* and were significantly (*p* < 0.01) higher for 60-, 120- and 180-day-old cheeses and 120-day-old cheeses, respectively (Table 6). Although some taste descriptors, such as “sour”, “bitter”, “sweet” or “salty”, were not influenced by the use of *L. salivarius* SP36 in cheese manufacture, “umami” descriptor scores were significantly higher in cheeses made with the *L. salivarius* strain (4.37 vs. 3.82, *p* < 0.01). There is a large number of factors that may exert an influence on the sensorial properties of cheeses, but among them, their microbial composition has a paramount relevance [39]. Overall, changes in the parameters cited above may be closely related to the impact of the strain in cheese maturation, most probably related to the proteolytic and lipolytic activities of the strain and its ability to participate in the biosynthesis of volatile compounds. The analysis of the genome of *L. salivarius* SP3 has already revealed the presence of a wide variety of genes potentially involved in such properties [15]. They are relevant traits since the flavour-forming potential is one of the LAB traits with the highest interest to the dairy industry [40]. Work is in progress to further elucidate the metabolic potential of such a strain for dairy applications.

## 4. Conclusions

*L. salivarius* SP36 proved to be a good candidate to be added as an adjunct culture for cheesemaking, since its use increased the formation of a variety of volatile compounds identified as potent odourants in several cheese varieties. As a result, the use of *L. salivarius* SP36 improved the cheese flavour by making it more intense and recovering typical sensorial notes of semi-hard and hard traditional cheeses made with raw sheep’s milk in Spain, such as the Manchego, Roncal, Zamorano and Idiazábal varieties. In addition, it had no negative effect on the rest of the cheese characteristics. Further studies would be very interesting to shed light on the metabolic pathways involved in the formation of volatile compounds, as well as to study *L. salivarius* SP36 functional properties in greater depth.

## Figures and Tables

**Table 1:** Counts of starter microorganisms (*L. lactis* and *S. thermophilus*) and *Lactobacillus* (mean ± SD log cfu/g) in the ewes’ milk cheeses made in this study.

Bacterial Species	Days	Type of Cheese	
		CS	LS
*L. lactis*	1	9.22 ± 0.02 ^a^	9.27 ± 0.02 ^a^
	60	8.58 ± 0.37 ^b^	8.28 ± 0.03 ^b^
	120	8.46 ± 0.02 ^b^	8.42 ± 0.08 ^b^
	180	8.02 ± 0.03 ^c^	8.06 ± 0.08 ^b^
	240	7.75 ± 0.26 ^d^	7.77 ± 0.23 ^c^
*S. thermophilus*	1	9.19 ± 0.16 ^a^	8.83 ± 0.31 ^a^
	60	8.57 ± 0.81 ^b^	8.69 ± 0.12 ^a^
	120	8.27 ± 0.13 ^b^	8.16 ± 0.11 ^a^
	180	7.76 ± 0.25 ^b^	7.27 ± 0.02 ^a^
	240	7.04 ± 0.23 ^c^	6.70 ± 0.11 ^a^
*Lactobacillus*	1	<3	8.72 ± 0.24 ^a^
	60	<3	6.54 ± 0.43 ^b^
	120	<3	6.47 ± 0.55 ^b^
	180	<3	6.85 ± 0.45 ^b^
	240	<3	6.85 ± 0.42 ^b^

^a–d^: Different letters in the same column indicate significant statistical differences for days of ripening (*p* < 0.05). *Lactobacillus* counts correspond to *L. salivarius*.

**Table 2:** Most abundant genera detected in the metataxonomic analysis performed for the ewes’ milk cheeses made in this study.

	Type of Cheese					
	CS			LS		
Genera	Day 1	Day 120	Day 240	Day 1	Day 120	Day 240
*Lactococcus*	79.20	81.31	62.86	77.81	78.86	68.72
*Streptococcus*	20.60	10.51	7.27	20.37	13.45	9.64
*Lactiplantibacillus*	0.00	7.22	18.61	0.00	4.53	17.84
*Ligilactobacillus*	0.03	0.02	0.03	1.68	1.12	2.24
Minor genera	0.17	0.95	1.23	0.14	2.03	1.56

**Table 3:** Esters ^a^ significantly affected by the use of *L. salivarius* SP36 as an adjunct culture in the ewes’ milk cheeses made in this study.

		Type of Cheese	
	Time (d)	CS	LS
Ethyl acetate	60	4.44 ± 0.47 ^a^	14.90 ± 9.58 ^b^
	120	7.46 ± 0.44 ^a^	18.09 ± 9.57 ^b^
	180	11.79 ± 2.44 ^a^	22.64 ± 6.97 ^b^
	240	20.57 ± 6.10 ^a^	52.27 ± 14.87 ^b^
Ethyl propanoate	60	ND ^a^	0.23 ± 0.02 ^b^
	120	0.63 ± 0.26 ^a^	1.23 ± 0.20 ^b^
	180	1.54 ± 0.36 ^a^	2.70 ± 0.29 ^b^
	240	2.13 ± 0.21 ^a^	4.98 ± 1.02 ^b^
Ethyl butanoate	60	4.84 ± 0.67	7.23 ± 1.86
	120	10.49 ± 1.51 ^a^	19.02 ± 4.13 ^b^
	180	28.95 ± 8.12 ^a^	37.56 ± 3.05 ^b^
	240	33.93 ± 4.24 ^a^	69.41 ± 4.60 ^b^
Ethyl valerate	60	ND	ND
	120	ND	ND
	180	ND ^a^	0.10 ± 0.01 ^b^
	240	0.11 ± 0.02 ^a^	0.28 ± 0.04 ^b^
Ethyl hexanoate	60	1.83 ± 0.24	2.60 ± 0.77
	120	4.57 ± 0.84 ^a^	7.17 ± 1.80 ^b^
	180	11.35 ± 1.39 ^a^	15.44 ± 3.24 ^b^
	240	14.70 ± 2.14 ^a^	28.50 ± 5.74 ^b^
Ethyl octanoate	60	ND ^a^	0.16 ± 0.07 ^b^
	120	0.33 ± 0.06 ^a^	0.61 ± 0.18 ^b^
	180	1.07 ± 0.23	1.63 ± 0.73
	240	1.21 ± 0.13 ^a^	2.30 ± 0.53 ^b^
Propyl acetate	60	0.40 ± 0.25	0.78 ± 0.25
	120	3.12 ± 0.62 ^a^	7.92 ± 3.08 ^b^
	180	6.42 ± 2.99 ^a^	20.35 ± 3.82 ^b^
	240	9.12 ± 3.13 ^a^	39.40 ± 13.75 ^b^
Propyl butanoate	60	ND ^a^	0.13 ± 0.07 ^b^
	120	0.73 ± 0.16 ^a^	1.87 ± 0.11 ^b^
	180	2.85 ± 0.53 ^a^	6.85 ± 0.57 ^b^
	240	4.21 ± 0.75 ^a^	12.12 ± 0.36 ^b^
Butyl acetate	60	0.17 ± 0.08 ^a^	0.90 ± 0.86 ^b^
	120	0.63 ± 0.22 ^a^	2.28 ± 1.78 ^b^
	180	1.79 ± 0.50 ^a^	5.13 ± 3.32 ^b^
	240	2.88 ± 0.76 ^a^	6.75 ± 2.81 ^b^
1-Methylpropyl acetate	60	ND	ND
	120	1.48 ± 0.81 ^a^	6.60 ± 5.46 ^b^
	180	2.45 ± 1.13	10.91 ± 10.76
	240	4.34 ± 2.83	14.25 ± 11.82 ^b^

^a^ Results are mean (n = 4) ± SD of duplicate determinations in two cheese trials, expressed as relative abundances to internal standard cyclohexanone. Means in the same row followed by different letters differ significantly (*p* < 0.01). ND, not detected.

**Table 4:** Aldehydes and alcohols ^a^ significantly affected by the use of *L. salivarius* SP36 as an adjunct culture in the ewes’ milk cheeses made in this study.

		Type of Cheese	
	Time (d)	CS	LS
3-Methyl butanal	60	0.20 ± 0.16	0.33± 0.05
	120	0.47 ± 0.22 ^a^	0.95 ± 0.18 ^b^
	180	0.80 ± 0.39	1.03 ± 0.10
	240	0.63 ± 0.08 ^a^	1.20 ± 0.16 ^b^
Ethanol	60	125.02 ± 9.86	164.67 ± 18.85
	120	121.73 ± 14.42	145.50 ± 17.18
	180	138.59 ± 25.01	158.20 ± 17.02
	240	130.64 ± 25.84 ^a^	221.30 ± 20.58 ^b^
1-Propanol	60	7.48 ± 3.13 ^a^	19.36 ± 11.65 ^b^
	120	32.27 ± 2.81 ^a^	65.70 ± 3.07 ^b^
	180	56.64 ± 1.88 ^a^	97.05 ± 4.73 ^b^
	240	52.63 ± 6.49 ^a^	116.35 ± 10.61 ^b^
1-Butanol	60	14.35 ± 2.73	23.57 ± 14.35
	120	17.94 ± 3.08	22.34 ± 5.88
	180	37.46 ± 6.50	42.64 ± 4.78
	240	55.33 ± 14.07 ^a^	87.83 ± 6.63 ^b^
1-Hexanol	60	4.93 ± 1.63	17.91 ± 18.53
	120	4.04 ± 1.55 ^a^	10.51 ± 7.82 ^b^
	180	8.20 ± 1.54 ^a^	13.38 ± 9.67 ^b^
	240	8.88 ± 2.14 ^a^	15.53 ± 7.83 ^b^
1-Octanol	60	ND	ND
	120	0.10 ± 0.03 ^a^	0.25 ± 0.16 ^b^
	180	0.28 ± 0.05 ^a^	0.54 ± 0.40 ^b^
	240	0.37 ± 0.11 ^a^	1.07 ± 0.79 ^b^
2-Pentanol	60	6.10 ± 2.40 ^a^	14.57 ± 2.96 ^b^
	120	19.76 ± 2.42 ^a^	33.29 ± 3.24 ^b^
	180	51.34 ± 10.38	48.25 ± 11.11
	240	77.16 ± 21.24	87.72 ± 16.01
1,2-Propanediol	60	0.39 ± 0.26 ^a^	1.56 ± 0.87 ^b^
	120	0.62 ± 0.26 ^a^	4.01 ± 2.12 ^b^
	180	2.94 ± 1.19 ^a^	8.17 ± 5.98 ^b^
	240	2.16 ± 0.16 ^a^	14.39 ± 2.86 ^b^
2-Furanmethanol	60	ND ^a^	0.31 ± 0.03 ^b^
	120	ND ^a^	0.33 ± 0.03 ^b^
	180	0.09 ± 0.01 ^a^	0.39 ± 0.04 ^b^
	240	0.10 ± 0.02 ^a^	0.46 ± 0.02 ^b^

^a^ Results are mean (n = 4) ± SD of duplicate determinations in two cheese trials, expressed as relative abundances to internal standard cyclohexanone. Means in the same row followed by different letters differ significantly (*p* < 0.01). ND, not detected.

**Table 5:** Carboxylic acids ^a^ significantly affected by the use of *L. salivarius* SP36 as an adjunct culture in the ewes’ milk cheeses made in this study.

		Type of Cheese	
	Time (d)	CS	LS
Propanoic acid	60	5.64 ± 1.38 ^a^	12.70 ± 2.05 ^b^
	120	59.80 ± 11.92 ^a^	91.62 ± 10.60 ^b^
	180	130.20 ± 22.29	158.48 ± 35.51
	240	131.06 ± 41.38 ^a^	186.09 ± 35.83 ^b^
2-Methyl propanoic acid	60	1.44 ± 0.37 ^a^	2.84 ± 1.66 ^b^
	120	2.13 ± 0.54	2.51 ± 0.69
	180	3.54 ± 0.12	3.58 ± 0.28
	240	4.48 ± 1.87 ^a^	6.22 ± 1.41 ^b^
2-Methyl butanoic acid	60	0.37 ± 0.24	0.54 ± 0.33
	120	0.47 ± 0.13	0.61 ± 0.24
	180	1.22 ± 0.24	0.87 ± 0.03
	240	1.09 ± 0.50 ^a^	1.70 ± 0.46 ^b^
3-Methyl butanoic acid	60	0.59 ± 0.46	0.76 ± 0.40
	120	0.53 ± 0.15	0.74 ± 0.35
	180	1.34 ± 0.27	0.98 ± 0.05
	240	1.17 ± 0.50 ^a^	1.88 ± 0.64 ^b^
Pentanoic acid	60	0.76 ± 0.10	0.78 ± 0.08
	120	1.11 ± 0.20	1.27 ± 0.12
	180	2.26 ± 0.57	1.99 ± 0.09
	240	2.85 ± 0.79 ^a^	3.69 ± 0.57 ^b^
Hexanoic acid	60	79.00 ± 1.87	75.02 ± 7.90
	120	107.09 ± 19.96	110.46 ± 12.34
	180	208.83 ± 44.43	175.16 ± 12.97
	240	229.55 ± 64.23 ^a^	293.97 ± 43.48 ^b^

^a^ Results are mean (n = 4) *±* SD of duplicate determinations in two cheese trials, expressed as relative abundances to internal standard cyclohexanone. Means in the same row followed by different letters differ significantly (*p* < 0.01).

**Table 6:** Sensory evaluation ^a^ of the cheeses made in this study.

		Type of Cheese	
	Time (d)	CS	LS
Odour intensity	60	5.54 ± 1.04	6.22 ± 1.07
	120	5.83 ± 1.55	6.75 ± 1.24
	180	5.89 ± 1.50	6.37 ± 1.65
	240	6.46 ± 1.61	6.58 ± 1.60
Odour quality	60	6.77 ± 1.12	7.16 ± 1.31
	120	6.89 ± 1.27	7.22 ± 1.31
	180	6.97 ± 1.00	7.41 ± 1.06
	240	7.04 ± 1.09	7.44 ± 1.27
Aroma intensity	60	6.11 ± 1.18 ^a^	6.86 ± 1.26 ^b^
	120	6.26 ± 1.04 ^a^	7.24 ± 1.22 ^b^
	180	6.19 ± 1.01 ^a^	7.11 ± 1.34 ^b^
	240	6.91 ± 1.31 ^a^	7.40 ± 1.26 ^b^
Aroma quality	60	6.92 ± 0.92	7.39 ± 1.29
	120	6.82 ± 1.21 ^a^	7.78 ± 0.96 ^b^
	180	7.19 ± 0.92	7.63 ± 1.30
	240	7.23 ± 1.01 ^a^	8.03 ± 1.18 ^b^
Taste intensity	60	6.05 ± 0.67 ^a^	7.25 ± 1.06 ^b^
	120	6.35 ± 1.07 ^a^	7.17 ± 1.00 ^b^
	180	6.69 ± 0.87 ^a^	7.46 ± 1.09 ^b^
	240	7.20 ± 1.29	7.49 ± 1.18
Taste quality	60	6.84 ± 1.06	7.61 ± 1.18
	120	6.49 ± 1.33 ^a^	7.45 ± 1.16 ^b^
	180	7.19 ± 0.81	7.60 ± 1.37
	240	7.27 ± 1.15	7.85 ± 1.36
Texture quality	60	7.11 ± 1.55	7.21 ± 1.42
	120	7.19 ± 1.22	6.81 ± 1.22
	180	7.00 ± 1.00	7.07 ± 1.08
	240	7.56 ± 1.26	7.63 ± 1.04
Colour quality	60	7.64 ± 1.86	7.79 ± 1.53
	120	8.09 ± 1.29	7.38 ± 1.54
	180	8.03 ± 1.32	7.60 ± 1.23
	240	7.69 ± 1.54	7.37 ± 1.29

^a^ Results are mean (n = 16) ± SD from 8 trained panelists on a 0–10 point scale of duplicate determinations in two cheese trials. Means in the same row followed by different letters differ significantly (*p* < 0.01).

## Data Availability

The original contributions presented in the study are included in the article/Supplementary Materials; further inquiries can be directed to the corresponding authors.

## Supplementary Materials

The following supporting information can be downloaded at https://www.mdpi.com/article/10.3390/foods13142296/s1, Figure S1: External and internal sensory descriptors of both cheese types (CS and LS) at days 60, 120, 180 and 240 of ripening; Table S1: Physical–chemical and colour parameters (mean ± SD) depending on the batch and days of ripening; Table S2: Texture parameters (mean ± SD) depending on the batch and days of ripening.
