# Supplementary material for: Application of Ligilactobacillus salivarius SP36, a Strain Isolated from an Old Cheese Seal, as an Adjunct Culture in Cheesemaking
Source: Foods. 2024 Jul 21;13(14):2296. doi: 10.3390/foods13142296 (PMC11276500; doi:10.3390/foods13142296)
Supplement: Supplementary file 1 [file foods-13-02296-s001.zip › foods-3040290-supplementary.pdf]

Supplementary Table S1. Physico-chemical and colour parameters (mean±SD) of the ewes' milk cheeses made in this study.

		Type of cheese		
**Parameter**	**Days**	**CS**	**LS**	
pH	1	5.09±0.05d	5.38±0.02a	*
	60	5.16±0.02c	5.23±0.01c	*
	120	5.23±0.04b	5.25±0.05b	ns
	180	5.24±0.07b	5.34±0.03b	ns
	240	5.36±0.04a	5.35±0.05b	ns
Fat (%)	1	34.21±0.30d	34.30±1.23c	ns
	60	38.58±0.34c	37.72±0.77b	ns
	120	39.34±0.71b	40.06±0.60a	ns
	180	40.61±0.65b	41.08±1.82a	ns
	240	41.99±0.03a	41.73±0.82a	ns
Protein (%)	1	23.45±0.35a	23.56±0.51b	ns
	60	23.38±0.46a	23.02±0.05b	ns
	120	23.79±0.62a	24.02±0.53b	ns
	180	24.45±0.12a	24.18±0.50ab	ns
	240	25.04±0.65a	25.77±0.30a	ns
Moisture (%)	1	40.51±0.72a	40.14±1.46a	ns
	60	32.09±0.88b	33.15±0.79b	ns
	120	30.73±1.52c	29.50±1.31bc	ns
	180	28.44±1.08c	28.18±0.52c	ns
	240	26.64±0.72d	26.39±0.87c	ns
NaCl (g)	1	0.73±0.02b	0.74±0.04c	ns
	60	1.37±0.05a	1.46±0.12b	ns
	120	1.32±0.03ab	1.39±0.02b	ns
	180	1.60±0.34a	1.58±0.45b	ns
	240	1.50±0.01a	1.89±0.11a	ns
Luminosity value (L)	1	89.78±0.94a	89.10±0.54a	ns
	60	82.58±0.31b	82.45±2.11b	ns
	120	81.73±0.93b	80.79±0.33b	ns
	180	79.84±0.66b	79.38±0.07b	ns
	240	75.75±1.30c	76.86±2.36b	ns
a*	1	-0.42±0.16a	-0.13±0.22a	ns
	60	-0.76±0.24b	-0.23±0.36a	ns
	120	-1.05±0.17b	-0.32±0.24a	ns
	180	-1.22±0.14b	-0.57±0.32a	ns
	240	-1.59±0.19c	-0.90±0.14a	ns
b*	1	12.19±1.24a	14.08±1.09a	ns
	60	12.99±0.22a	14.86±2.20a	ns
	120	14.70±1.46a	16.30±0.25a	ns
	180	14.27±1.99a	15.30±0.54a	ns
	240	14.89±2.65a	15.86±0.94a	ns

CS: cheese made with commercial starter; LS: cheese made with commercial starter and *L. salivarius*. a-c: Different letters in the same column indicate significant statistical differences for days of ripening ($p < 0.05$).*: Significant differences between CS and LS cheeses ($p < 0.05$). ns: No significant differences between CS and LS cheeses ($p < 0.05$).

Supplementary Table S2. Texture parameters (mean±SD) of the ewes' milk cheeses made in this study.

		Type of cheese		
**Parameter**	**Days**	**CS**	**LS**	
Hardness (N)	1	8,01±0.52a	8,66±1.91b	ns
	60	13,46±0.73a	14,61±0.14a	ns
	120	11,99±1.88a	10,28±0.85ab	ns
	180	10,52±1.37a	10,45±1.77ab	ns
	240	15,06±4.72a	11,88±0.37ab	ns
Adhesiveness (N*s)	1	-0,06±0.03a	-0,06±0.05a	ns
	60	-0,09±0.08a	-0,09±0.09a	ns
	120	-0,05±0.02a	-0,03±0.02a	ns
	180	-0,05±0.02a	-0,01±0.00a	ns
	240	-0,01±0.01a	-0,02±0.02a	ns
Cohesiveness	1	0,78±0.00a	0,78±0.00a	ns
	60	0,57±0.02b	0,38±0.04b	ns
	120	0,40±0.02c	0,37±0.06b	ns
	180	0,28±0.01d	0,26±0.05b	ns
	240	0,23±0.00d	0,25±0.03b	ns
Gumminess	1	6,26±0.40b	6,74±1.48a	ns
	60	7,65±0.70a	5,60±0.48ab	ns
	120	4,75±0.44bc	3,85±0.89ab	ns
	180	2,90±0.24c	2,68±0.12b	ns
	240	3,47±1.11c	2,96±0.55b	ns
Chewiness (N)	1	6,26±0.40bc	6,74±1.48a	ns
	60	7,65±0.70a	5,94±0.83a	ns
	120	4,92±0.44bc	4,15±1.00a	ns
	180	3,62±0.25c	3,61±1.04a	ns
	240	5,43±1.11bc	4,02±0.20a	ns
Springiness	1	1,00±0.00c	1,00±0.00a	ns
	60	1,00±0.00c	1,06±0.06a	ns
	120	1,03±0.05bc	1,10±0.01a	ns
	180	1,24±0.11bc	1,36±0.44a	ns
	240	1,60±0.04a	1,38±0.20a	ns
Resilience	1	0,49±0.01b	0,47±0.01a	ns
	60	0,23±0.00b	0,27±0.09a	ns
	120	0,21±0.07b	0,32±0.01a	ns
	180	0,50±0.19b	0,69±0.71a	ns
	240	1,00±0.02a	0,73±0.27a	ns

CS: cheese made with commercial starter; LS: cheese made with commercial starter and *L. salivarius.* a-b: Different letters in the same column indicate significant statistical differences for days of ripening ($p < 0.05$).*: Significant differences between CS and LS cheeses ($p < 0.05$). ns: No significant differences between CS and LS cheeses ($p < 0.05$).

Figure S1. External and internal sensory descriptors of both cheese types (CS and LS) at days 60, 120, 180 and 240 of ripening.

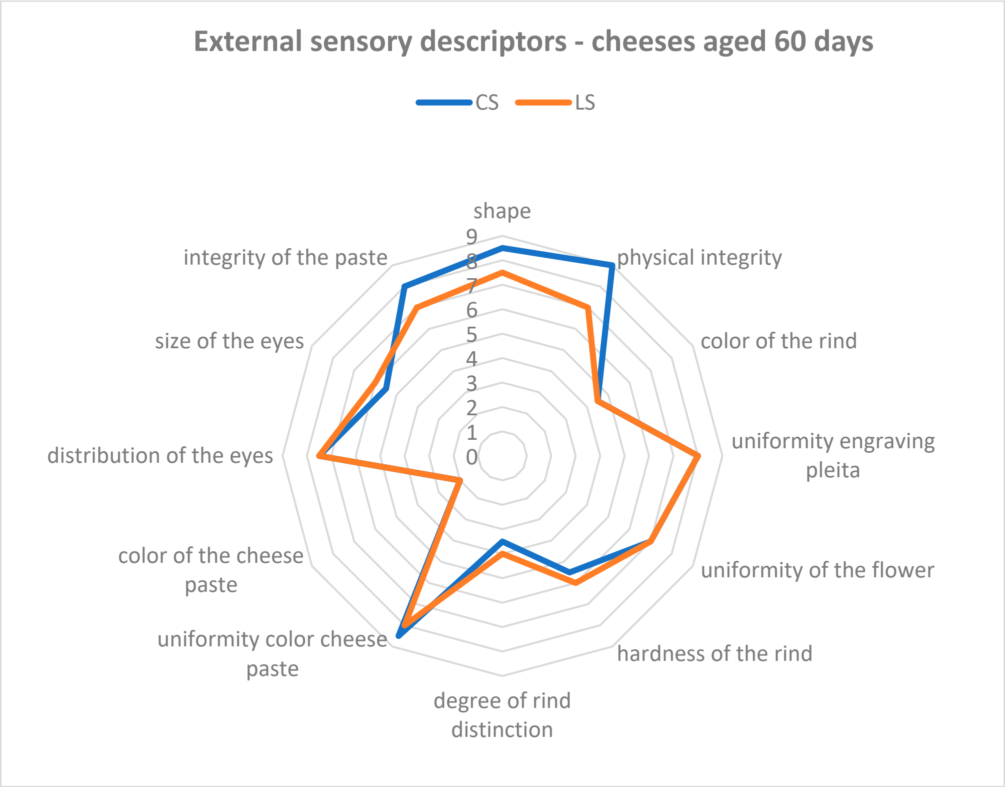

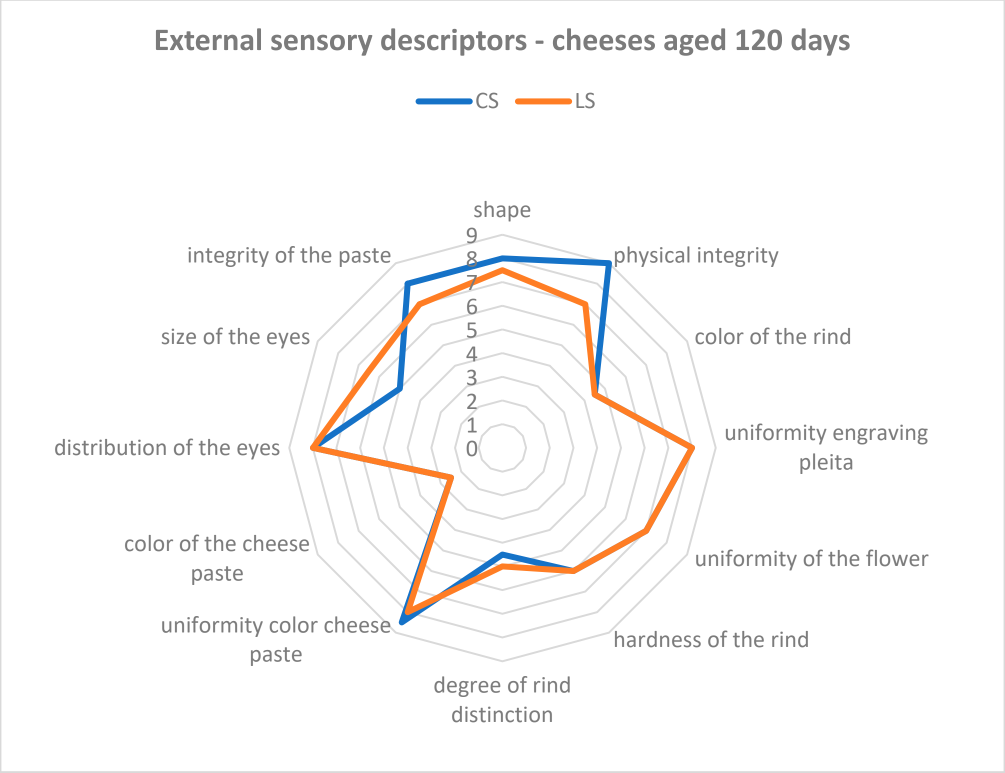

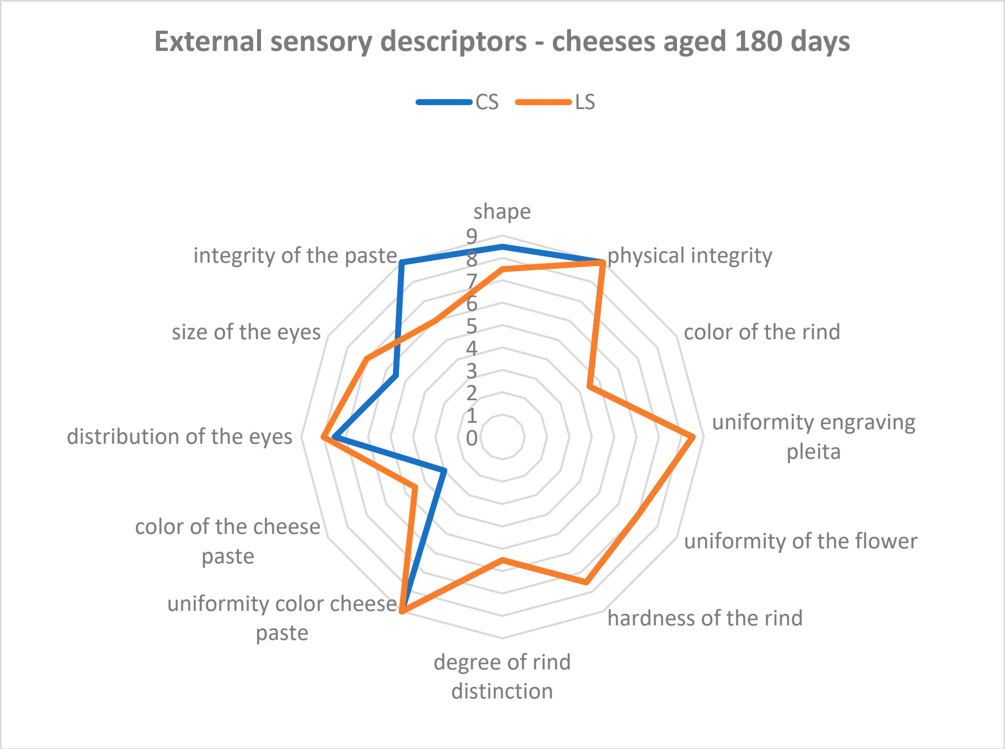
External sensory descriptors - cheeses aged 180 days
CS
LS
shape
physical integrity
color of the rind
uniformity engraving pleita
uniformity of the flower
hardness of the rind
degree of rind distinction
uniformity color cheese paste
color of the cheese paste
distribution of the eyes
size of the eyes
integrity of the paste
9
8
7
6
5
4
3
2
1
0

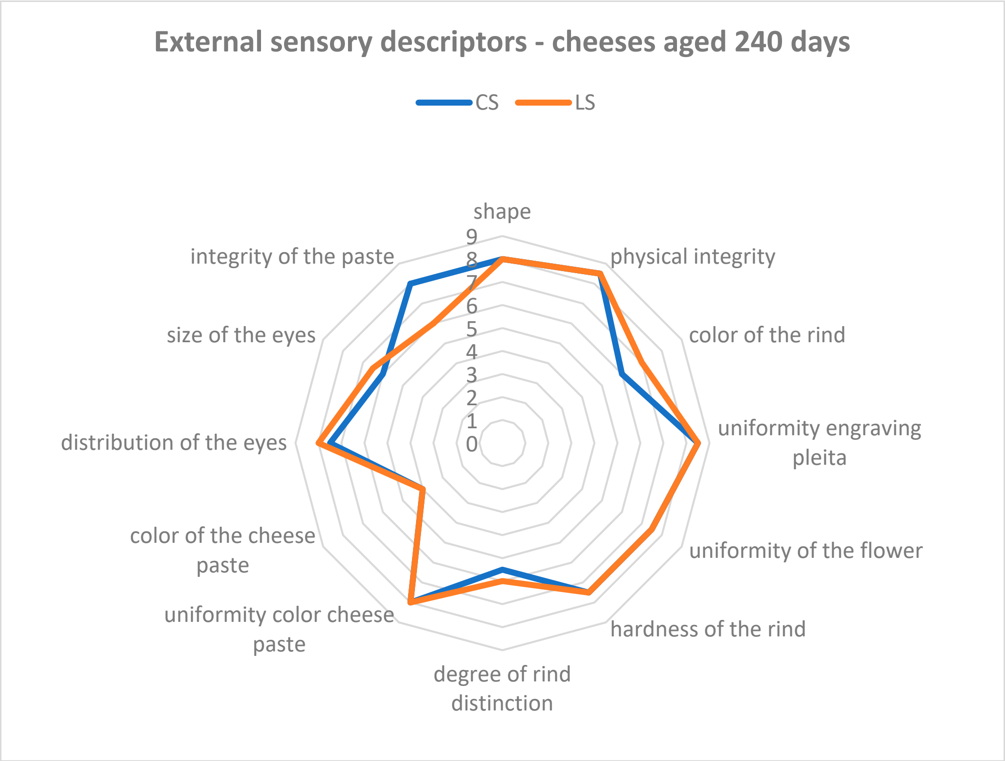
External sensory descriptors - cheeses aged 240 days
CS
LS
shape
physical integrity
color of the rind
uniformity engraving pleita
uniformity of the flower
hardness of the rind
degree of rind distinction
uniformity color cheese paste
color of the cheese paste
distribution of the eyes
size of the eyes
integrity of the paste
9
8
7
6
5
4
3
2
1
0

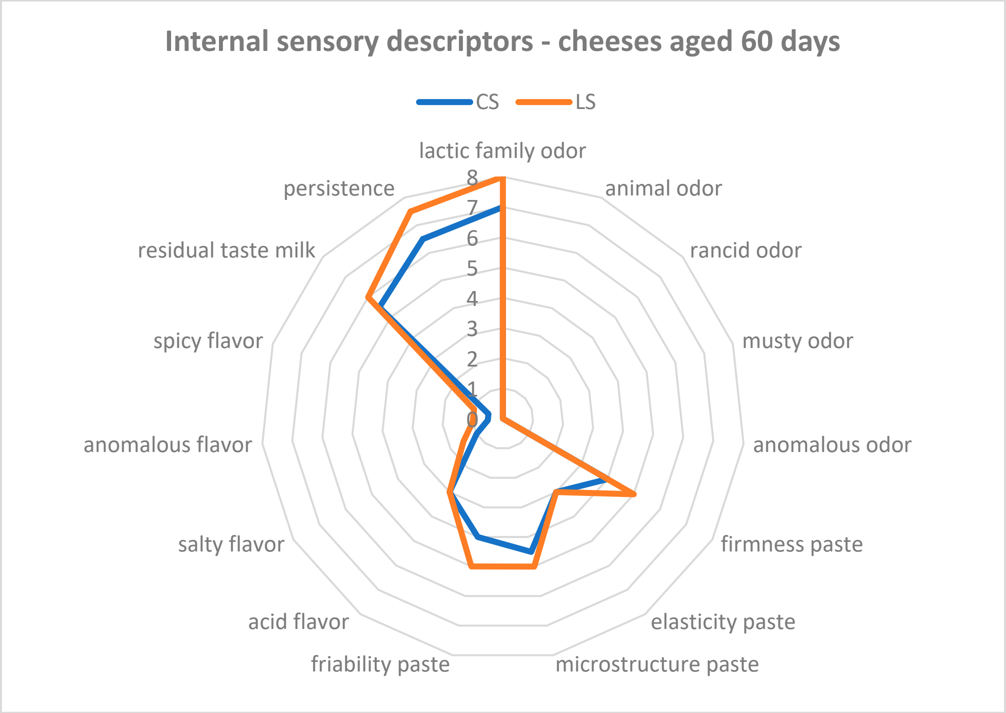
Internal sensory descriptors - cheeses aged 60 days
CS
LS
lactic family odor
animal odor
rancid odor
musty odor
anomalous odor
firmness paste
elasticity paste
microstructure paste
friability paste
acid flavor
salty flavor
anomalous flavor
spicy flavor
residual taste milk
persistence
8
7
6
5
4
3
2
1
0

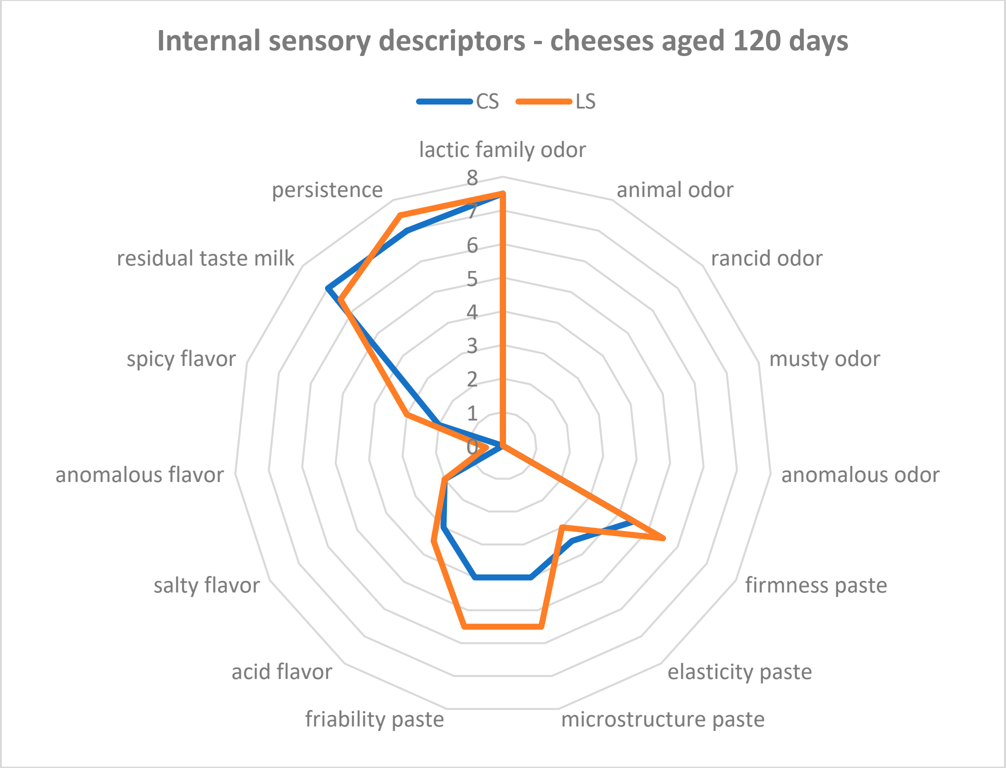
Internal sensory descriptors - cheeses aged 120 days
CS
LS
lactic family odor
animal odor
rancid odor
musty odor
anomalous odor
firmness paste
elasticity paste
microstructure paste
friability paste
acid flavor
salty flavor
anomalous flavor
spicy flavor
residual taste milk
persistence
8
7
6
5
4
3
2
1
0

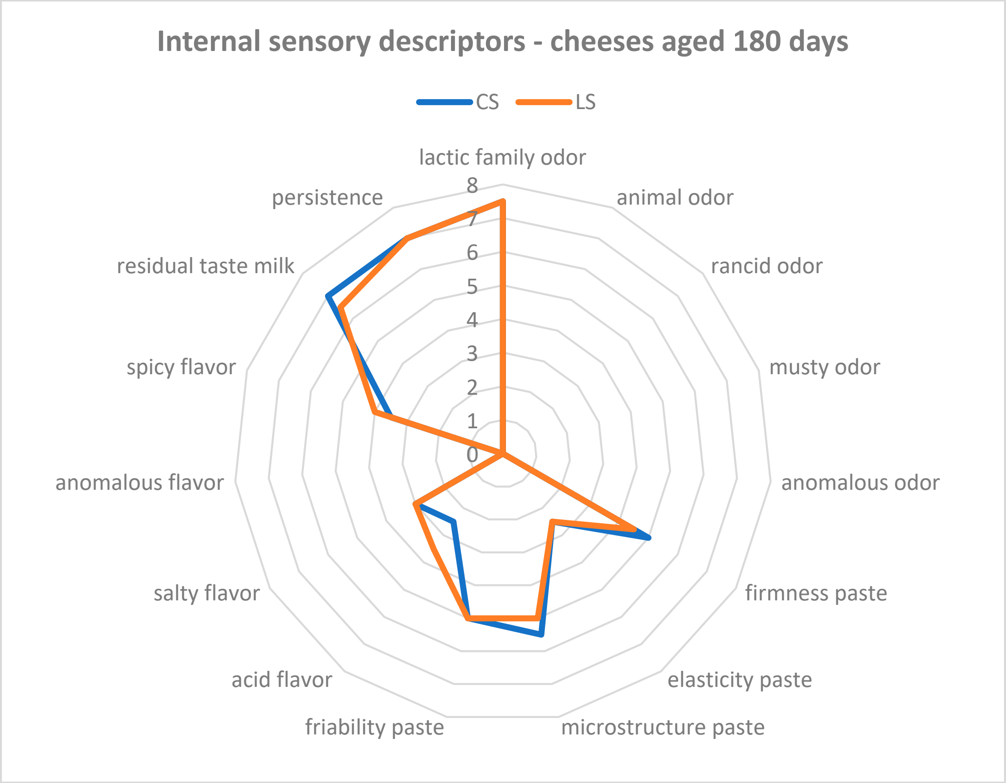
Internal sensory descriptors - cheeses aged 180 days
CS
LS
lactic family odor
animal odor
rancid odor
musty odor
anomalous odor
firmness paste
elasticity paste
microstructure paste
friability paste
acid flavor
salty flavor
anomalous flavor
spicy flavor
residual taste milk
persistence
8
7
6
5
4
3
2
1
0

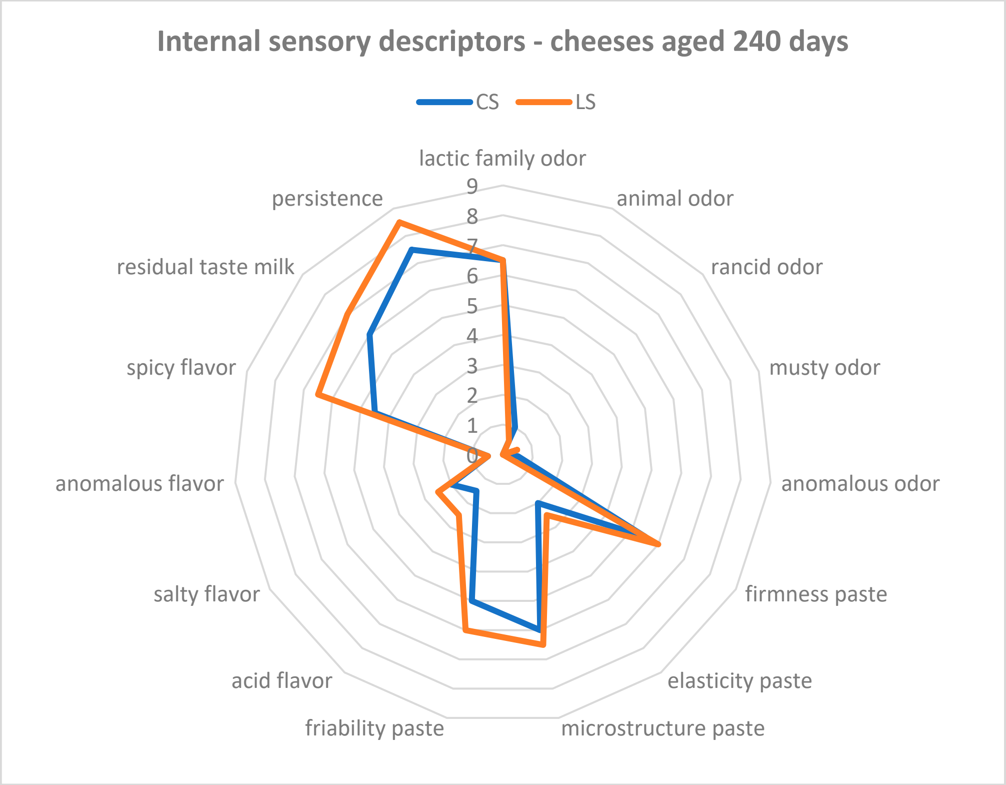
Internal sensory descriptors - cheeses aged 240 days
CS
LS
lactic family odor
animal odor
rancid odor
musty odor
anomalous odor
firmness paste
elasticity paste
microstructure paste
friability paste
acid flavor
salty flavor
anomalous flavor
spicy flavor
residual taste milk
persistence
9
8
7
6
5
4
3
2
1
0
